# Targeting antimicrobial restriction: outcomes of pharmacist-led stewardship interventions at the university hospital Salzburg

**DOI:** 10.1007/s15010-025-02604-x

**Published:** 2025-07-09

**Authors:** Eva Past, Laura Hartmann, Robert Zimmermann, Georg Zimmermann, Markus Wallner, Lisa Walter, Ulrike Porsche, Jan Marco Kern

**Affiliations:** 1 Department of Clinical Pharmacy and Drug Information, Landesapotheke Salzburg, University Hospital Salzburg, Salzburg, Austria; 2 Institute of Pharmacy, Paracelsus Medical University, Salzburg, Austria; 3 Independent Researcher, Traunstein, Germany; 4 Department of Artificial Intelligence and Human Interfaces, Faculty of Digital and Analytical Sciences, Paris Lodron University, Salzburg, Austria; 5 Research Programme Biomedical Data Science, Paracelsus Medical University, Salzburg, Austria; 6 Institute of Clinical Microbiology and Hygiene, Paracelsus Medical University, University Hospital Salzburg, Salzburg, Austria

**Keywords:** Antimicrobial stewardship, Post-prescription authorization, Pharmacist-led interventions, Drug-related problems

## Abstract

**Purpose:**

Antimicrobial overuse and misuse remain critical challenges. This study examined pharmacist-led post-prescription interventions targeting restricted antimicrobials in a university hospital, identifying underlying drug-related problems (DRPs), their clinical relevance, economic impact and characteristic patterns of inappropriate use.

**Methods:**

A retrospective observational analysis (January– December 2022) was conducted at the Salzburg State Hospitals using routine data of pharmacist-led interventions on restricted antimicrobials. DRPs and intervention types were categorized using validated criteria. Clinical relevance was independently assessed through an external survey, and interrater reliability was determined to ensure consistency in classification and evaluation. Potential cost savings and acceptance rates of the pharmaceutical interventions were assessed.

**Results:**

A total of 3897 restricted antimicrobial prescriptions were analyzed, with 11.7% (456) showing at least one DRP in 366 patients. The majority of DRPs (80.2%) exhibited marked clinical relevance, mainly due to non-conformance with guidelines (27.4%), unclear indication (27.2%), and the need for patient or drug monitoring (12.5%). Broad-spectrum agents linezolid (25.0%), meropenem (24.1%), ciprofloxacin (15.8%), and piperacillin-tazobactam (8.8%) accounted for nearly 74% of all DRPs. DRP-related interventions aimed at optimizing PK/PD parameters (30.6%), treatment discontinuation (28.1%), and de-escalation (17.9%). The acceptance rate of interventions was high (82.7%). A cost reduction potential was identified in 89.7% of interventions, saving €180,420 in avoided drug expenses.

**Conclusion:**

Pharmacist-led post-prescription interventions within an established AMS program effectively identified clinically relevant misuse of restricted antimicrobials. Targeted actions on key agents enable high-impact optimization, supported by strong acceptance and cost-saving potential - thereby enhancing stewardship efforts, guiding improvements in diagnostics, and prescribing behavior.

## Introduction

Reducing the overuse and misuse of antimicrobial agents remains a significant challenge in combating the emergence of resistance and minimizing patient harm. In this context, formulary restrictions of antimicrobials have long been a fundamental component of antimicrobial stewardship (AMS) programs in hospital settings [1]. Clinical pharmacists play a pivotal role by reviewing restricted prescriptions, consulting infectious disease (ID) specialists, and authorizing approved treatments [2]. Pharmacist-led pre-authorizations are widely implemented [3] and have shown to reduce inappropriate use of anti-infectives, and to optimize both empirical and targeted antimicrobial therapies while reducing associated costs [4]. Restricted antimicrobials typically include broad-spectrum agents (e.g., carbapenems, anti-pseudomonal beta-lactams) and those with significant toxicity or cost. Additionally, limitations on newly marketed agents help prevent resistance from the outset. Thus, a designated list of restricted antimicrobials should be an integral part of every hospital formulary [5]. Beyond their direct impact, these restrictions can facilitate behavior change interventions to reach AMS objectives such as improved prescribing practices and guideline adherence [6, 7]. They also serve an educational purpose, requiring prescribers to adhere to AMS protocols and justify their antimicrobial choices [8].

This study assessed the impact of pharmacist-led post-prescription interventions on anti-infective use by identifying key agents of concern, detecting drug-related problems (DRPs), and evaluating the clinical relevance of interventions. It also examined systemic weaknesses in managing restricted antimicrobials within our university hospital in an established AMS-setting. Additionally, cost savings from these interventions were estimated.

## Methods

This retrospective observational study involved a secondary data analysis of pharmaceutical interventions, complemented by an online survey to validate their clinical impact. Conducted at Salzburg State Hospitals (Salzburger Landeskliniken, SALK), a 2,000-bed network comprising the University Hospital of Salzburg (tertiary care, two sites in the city of Salzburg with campus CDK (Christian-Doppler-Klinik) and campus LKH (Landeskrankenhaus) and three regional hospitals in Hallein, St. Veit, and Tamsweg, the study was supported by the central pharmacy (Landesapotheke Salzburg), responsible for drug procurement, compounding, and clinical pharmacy services. The network’s AMS program is managed by a multidisciplinary team, including an infectious diseases (ID) consultant (0.5 full-time equivalent, FTE), three clinical microbiologists (1 FTE), an infection prevention specialist (1 FTE), and three AMS-trained clinical pharmacists (0.6 FTE). The team updates antimicrobial guidelines, accessible on the hospital intranet, and supports them with audits and feedback on antibiotic use and resistance. The anti-infective treatment guidelines and AMS policies are the same in the whole hospital network (SALK).

**Fig. 1:**
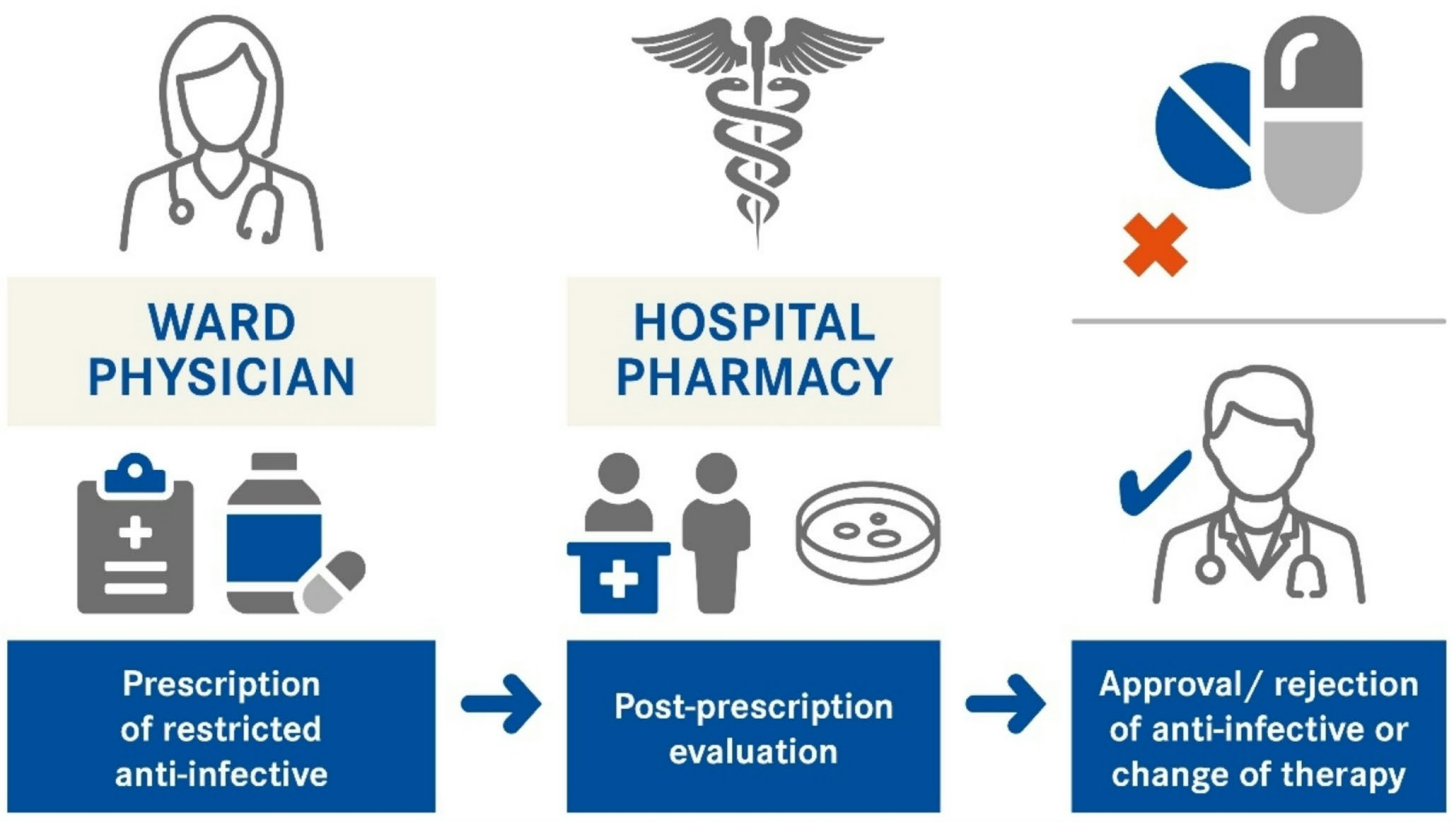
Post-prescription algorithm. A restricted antimicrobial list including carbapenems, piperacillin/tazobactam, linezolid, advanced-generation cephalosporins, fluoroquinolones, and most antifungals (except fluconazole) requires AMS pharmacist review and may involve infectious disease or microbiologist consultation before dispensing

### Prescription review data

A dataset entered between January 2 and December 30, 2022 by three AMS pharmacists was retrospectively analyzed. The dataset comprised routine data from pharmaceutical interventions during the prescription review of restricted antimicrobials (Monday to Friday, 8 am to 4 pm) based on the AWaRe (access, watch, reserve) principles by the World Health Organization (WHO) concerning anti-infective agents [9]. Data from the out-of-hours pharmacy services were not analyzed. The reviewed antimicrobials included selected antibiotics from the WHO Reserve and Watch list, as well as certain antifungals and antivirals (Table 1). The AMS team reviewed the list annually, considering resistance trends, consumption data, and official guidelines like the EMA’s recommendation for fluoroquinolones as second-line agents. New agents, such as novel cephalosporin/beta-lactamase inhibitor combinations, were automatically added to the restricted list. The hospital network’s therapeutics committee must approve the list and any amendments.

**Table 1:** List of restricted antimicrobial agents for pharmacist review prior to dispensing

Antimicrobial group	Substances
Antibiotics	Carbapenems: ertapenem, imipenem-cilastatin, meropenem Advanced generation cephalosporines: cefiderocol, ceftaroline Novel generation cephalosporines: ceftazidime-avibactam, ceftolozane-tazobactam Fluorochinolones: ciprofloxacin, delafloxacin, levofloxacin, moxifloxacin, prulifloxacin Glycolipo-peptides: dalbavancin, daptomycin, oritavancin Other antibiotic agents: aztreonam-avibactam, colistine, fidaxomicin, linezolid, tigecyline
Antifungals	Azoles: isavuconazole, posaconazole, voriconazole Echinocandins: anidulafungin, caspofungin, micafungin Polyenes: amphotericine B liposomal
Antivirals	Valganciclovir

In 2022, prescriptions were performed either electronically (30%) or paper-based (70%) depending on the clinical department and hospital site as the interfaces of the electronic prescribing system (ORBIS Medication™) and the drug dispensing software (SAP™) were incompatible. Therefore, the prescriptions of restricted antimicrobials and other agents outside the hospital formulary had to be faxed from the ward to the pharmacy until 10:30 am by nursing or clerical staff.

AMS pharmacists then reviewed each prescription according to a standard operating procedure (SOP), which involved assessing the indication, checking ID consultations, antimicrobial susceptibility tests (AST), and adherence to local AMS guidelines. AMS pharmacists also performed checks for drug interactions, appropriate dosing (considering patient weight, renal function, and therapeutic drug monitoring, TDM), using clinical data from the electronic health record (EHR) system used across the entire hospital network. After this review, the antimicrobial was either approved and dispensed to the ward or further consultation with the ward physician or ID specialist was sought for clarification. The prescription was then modified (e.g., dosage or route adjusted) or rejected (Fig. 1). Furthermore, there were no official antimicrobial delivery limits, but the review process considered the proposed therapy duration from the infectious disease physician’s recommendation. The service required 0.25 FTE of a pharmacist.

### Drug-related problems and pharmaceutical interventions

Any identified drug-related problem (Table 2) was discussed with the prescriber, another ward clinician, or an ID physician. Each DRP was followed by a pharmaceutical intervention (Table 3). Both the DRPs and the intervention were categorized using an adapted version of the Société Française de Pharmacie Clinique (SFPC) criteria [10]. AMS pharmacists recorded anonymized patient data (age, gender, treating department) and summarized each intervention. The time from DRP identification to intervention was categorized as within 15 min, 15–60 min, or over an hour. Outcomes were documented within one or two days. Direct cost-savings were documented when a restricted antimicrobial was not dispensed at all or if fewer items were dispensed. The calculations were based on the number of non-dispensed items (e.g. three instead of ten vials for a three-day treatment– the cost of the seven vials was regarded as a cost saving). In case an IV-to-oral switch was conducted, cost saving was calculated as the difference between the cost of the ordered IV medication and the equivalent amount of oral treatment. For example, ciprofloxacin 400 mg IV (10 ampoules for 5 days, 400 mg twice a day) was compared to ciprofloxacin 500 mg tablets (10 tablets for 5 days, 500 mg twice a day).

**Table 2:** Categories of drug-related problems identified during post-prescription review of restricted antimicrobials referred to [10]

Drug-related problem	Description
Non-conformance (guideline, ID consultation, AST)	Non conformity of a drug compared to a guideline, present statement of an ID consultation or present microbiological results/AST
Untreated indication (need for an anti-infective drug)	Need for an anti-infective drug due to - Valid indication identified without drug prescription - Untreated symptom - Omission of an antibiotic dose during ward transfer
Dosage too low	Dosage too low
Dosage too high	Dosage too high including drug duplicate (same active substance prescribed several times)
Drug without clear indication	No valid indication for a drug
Drug-drug interaction	Drug-drug interference with increased risk of side effects Reduction of the effectiveness of a drug (risk of therapeutic failure)
Adverse drug reaction	Adverse drug event upon appropriate dosage
Inappropriate drug application	Appropriate substance and dosage but change of admission route would have been more effective or less costly Inappropriate drug formulation Inappropriate timing of the administration
Need for patient/drug monitoring	Inappropriate drug monitoring (missing TDM, laboratory or clinical parameters)
Incomplete information	Incomplete information in the substance ordering form
Administrative issue	Organizational aspect of the ordering or approval process

*AST antimicrobial susceptibility test; ID infectious disease; TDM therapeutic drug monitoring

**Table 3:** Categories of pharmaceutical interventions used during post-prescription review of restricted antimicrobials referred to [10]

Intervention	Description (examples)
Addition of a new drug	A new drug is added to the ongoing treatment
Treatment discontinuation	A drug is discontinued without a substitution
De-escalation	Change to a narrower-spectrum anti-infective based upon cultures
Adjustment of treatment duration	Determination of a specific treatment duration (e.g. according to guidelines)
IV-PO switch	Switch to oral therapy with high bioavailability in absence of contraindication
Optimization of PK/PD parameters	Optimization of drug dosing (according to organ function, drug level), dosage interval or duration of infusion
Administrative information	Information about the drug dispensing process or availability of drugs

*IV-PO intravenous to oral; PK/PD pharmacokinetic/pharmacodynamic

### Clinical relevance of pharmaceutical interventions

Drug prices were sourced from the hospital drug information system (eMedicInfo™). Savings were calculated based on national pharmacy purchase prices. Interventions with potential indirect cost savings (e.g., fewer adverse events or shorter stays) were categorized but not financially quantified.

The clinical relevance of each pharmaceutical intervention had been graded by the AMS pharmacist according to the Hatoum scale (Table 4), ranging from not relevant to extremely relevant [11].

**Table 4:** Levels of clinical relevance of interventions of pharmaceutical interventions referred to the Hatoum scale [11]

Level of clinical relevance	Definition	Example
Not relevant	For informational purposes only, not an improvement to therapy	• Information on order number • Order or delivery information
Slightly relevant	Patients and staff can benefit from the recommendation	• Switching to a cheaper but equally effective preparation (‘aut idem’) • Clarification of an unreadable entry in the patient chart or request
Relevant	Recommendation substantially improves the patient’s drug therapy	• Dose adjustment • Notification of the presence of a relative contraindication • Monitoring of an interaction that has not yet occurred (e.g. possible QT prolongation) • Inadequately controlled chronic disease (arterial hypertension, diabetes mellitus, hyperlipoproteinaemia) with specific recommendation of therapy optimization and/or change of substance • Recommendation to discontinue unnecessary anti-infective therapy • Risk of electrolyte imbalance with recommendation of change of substance, IV-PO switch
Highly relevant	Recommendation prevents or resolves an acute risk to the patient	• Existing contraindication • Lack of TDM or lack of dose adjustment with TDM available • Potentially dangerous drug interaction with existing symptoms • Untreated infection (threatening)
Extremely relevant	Recommendation to avert an acutely life-threatening situation for the patient	• Severe electrolyte imbalance • Life-threatening serotonergic syndrome • Risk of life-threatening cardiac arrhythmias (manifest long QT syndrome, known torsade de point tachycardia)

*IV-PO intravenous to oral; TDM therapeutic drug monitoring

### Questionnaire survey and expert team

To assess the external validity of pharmacists’ interventions, a questionnaire survey was conducted with seven AMS experts in internal medicine, infectious diseases, or clinical pharmacy from Austria and Germany. Participants, who were invited via email, agreed to participate. The survey included study details, demographic questions, and AMS experience, followed by 30 randomly selected patient cases (without clinical relevance levels). Experts evaluated the clinical relevance of each intervention using the Hatoum scale [11], which has been previously applied in other Austrian and international studies for ranking the clinical relevance of pharmaceutical interventions [12, 13].

### Data analysis

The 2022 dataset of pharmaceutical interventions was analyzed descriptively using Python programming language (Python Software Foundation, www.python.org/) and the pandas package for data analysis (https://pandas.pydata.org/). Nominal data were presented as frequencies and percentages, while central tendencies were given as median and interquartile range (IQR) [14]. Interrater reliability and agreement were assessed using Fleiss’s kappa, (weighted) Cohen’s kappa, and Gwet’s AC1 and AC2 coefficients [15] with the statistical software package R Version 4.3.1. (www.r-project.org).

Fleiss’ kappa generalizes Cohen’s kappa for more than two raters and measures the degree of exact agreement. In contrast, weighted Cohen’s kappa accounts not only for exact matches but also for similar ratings. Gwet’s coefficients represent more recent developments in interrater reliability analysis, incorporating less restrictive assumptions about chance agreement. As a result, they generally yield higher values than Cohen’s and Fleiss’ kappa, particularly in the presence of unequal marginal distributions. AC1 considers only exact matches, whereas AC2 incorporates similar ratings using ordinal weighting. All these coefficients share a common interpretation: a maximum value of 1 indicates perfect agreement, while a value of 0 reflects agreement due to chance.

To determine inter-rater reliability for the experts, the median of the pairwise coefficients for all possible rater pairs was calculated. For the evaluation of the agreement between the expert team and the original clinical relevance rating, the median of the ratings by the expert team was taken as the value representing the expert opinion, which was then assessed with respect to agreement with the original rating by using Cohens Kappa, Weighted Cohens Kappa, AC1 and AC2.

All analyses were conducted both for the entire expert team and separately by professional group (clinical pharmacists and physicians). Ratings classified as “not evaluable” were treated as a separate category in the calculations of Cohen’s kappa, Fleiss’ kappa, and AC1, whereas they were considered missing values in AC2. For weighted Cohen’s kappa, all cases labelled “not evaluable” were excluded from the analysis, as their distances to other categories could not be meaningfully determined.

## Results

### Retrospective observational study

The 2022 data set comprised 3,897 prescriptions of restricted antimicrobials. The majority of patients (*n* = 380, 93.6%) were from the two largest hospitals in the network (Campus CDK and LKH). In 11.7% (*n* = 456 attributed to 366 patients) of the analyzed prescriptions, at least one DRP was identified and a corresponding intervention was proposed. Most of the patient cases associated with DRPs were assessed within 15 min (*n* = 430; 94.3%), while 26 cases (5.7%) required more than 15 min, with a maximum workup time of 60 min. The majority of the 366 patients with DRPs were male (*n* = 218; 59.6%) and older than 65 years (*n* = 256; 69.9%) with a median age of 72.5 years. Table 5 shows the demographic information for the patients with DRPs within the context of restricted antimicrobials.

**Table 5:** Demographic details of patients (*n* = 366) with at least one DRP-related prescription of a restricted antimicrobial agent

Patient characteristic	Patients (*n* = 366) *n* (%)
**Gender– *n*** **(%)**	
Male	218 (59.6)
Female	148 (40.4)
**Age - years**	
Median (interquartile range)	72.5 (62–81)
Range	7–97
Patients < 65 years– *n* (%)	110 (30.1)
Patients ≥ 65 years	256 (69.9)
**Patients per department**	
Internal medicine	211 (57.6)
Surgery	131 (35.8)
Other (e.g. dermatology, urology)	24 (6.6)

*DRP drug-related problem

### Clinical relevance of drug-related problems

**Fig. 2:**
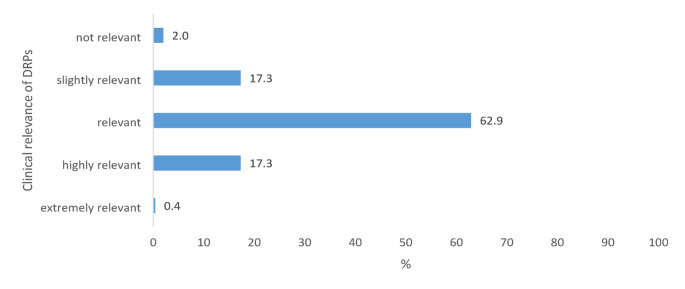
Rate of clinical relevance (in %) of drug-related problems (DRP, *n* = 456) identified within the post-prescription process of restricted antimicrobials

The vast majority of the 366 patients presented with a single DRP (83.6%, *n* = 306), while 11.7% (*n* = 43) of patients presented with two DRPs. Analysis of the DRPs showed high clinical relevance (CR) in 80.2% with most categorized as “relevant” (62.9%, *n* = 287 out of 456 DRPs) or “highly relevant” (17.3%, *n* = 79) (Fig. 2). Only two DRPs (0.4%) were categorized as ‘extremely relevant’, while the categories ‘slightly relevant’ and ‘not relevant’ accounted for 17.3% and 2% of DRPs (*n* = 79 and *n* = 9, respectively).

### Reasons for a drug-related problem

**Fig. 3:**
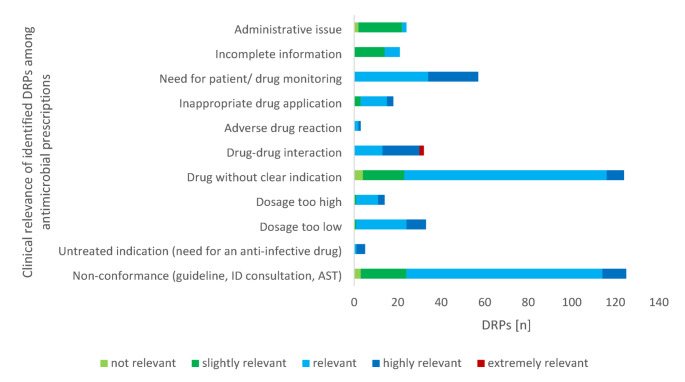
Clinical relevance of identified reasons for drug-related problems (in total *n* = 456) among antimicrobial prescriptions within the post-prescription process of restricted agents AST antimicrobial susceptibility testing; DRP drug-related problem; ID infectious diseases

The main reasons for a DRP related to an antimicrobial agent (Fig. 3) were ‘non-conformance of anti-infective administration with guidelines, existing infectious disease advice or AST’ (27.4% of all DRPs, n = 125), ‘drug without clear indication’ (27.2%, n = 124) and ‘need for patient or drug monitoring’ (12.5%, n = 57). Sub-analysis of these categories revealed the highest rates of CR-categories “relevant” or “highly relevant”. The category ´drug-drug-interaction´ was the only one that included two cases classified as “extremely relevant” and also showed a substantial rate of clinically relevant (n = 13) or highly relevant (n = 17) DRPs. In addition, ‘dosage’ aspects (both too high and too low) were also of great importance, together accounting for 10.3% of DRPs. In contrast, administrative aspects of the prescription or incomplete information in the substance application played a minor role.

### Key anti-infective agents of concern

**Fig. 4:**
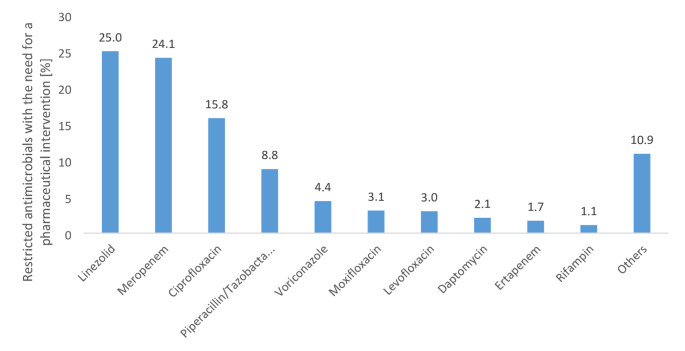
Rate of restricted antimicrobials with the need for a pharmaceutical intervention within the post-prescription process (*n* = 456)

Our analysis demonstrated that four broad-spectrum anti-infective substances accounted for 73.7% of all interventions: linezolid (25.0%, *n* = 114), meropenem (24.1%, *n* = 110), ciprofloxacin (15.8%, *n* = 72), and piperacillin-tazobactam (8.8%, *n* = 40) (Fig. 4). Voriconazole required 4.4% (*n* = 20) of consultations, while the quinolones levofloxacin and moxifloxacin collectively represented 5.9% (*n* = 27).

**Fig. 5:**
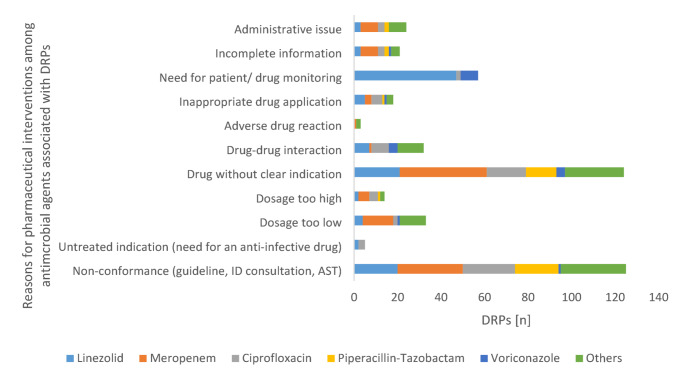
Detailed analysis of reasons for a pharmaceutical intervention among key antimicrobial agents associated with a drug-related problem within the post-prescription process (*n* = 456) AST antimicrobial susceptibility testing; DRP drug-related problem; ID infectious diseases

In the detailed analysis of the reasons for DRPs, certain patterns stood out prominently (Fig. 5): linezolid was the leading contributor to “need for patient/drug monitoring”, accounting for 82.4% of DRPs within this category (*n* = 47), followed by voriconazole at 14% (*n* = 8). Therapeutic drug monitoring (TDM) is available for both of these substances in our hospitals.

The DRP category “drug without clear indication” was predominantly associated with the substances meropenem (32.2%, *n* = 40), linezolid (16.9%, *n* = 21), ciprofloxacin (14.5%, *n* = 18), and piperacillin-tazobactam (11.3%, *n* = 14). The very same agents also represented the majority of cases in the category “non-conformance with guidelines, ID consultation, or AST” with meropenem accounting for 24.0% of DRPs (*n* = 30), followed by ciprofloxacin (19.2%, *n* = 24), as well as linezolid and piperacillin-tazobactam (each 16.0%, each *n* = 20). Meropenem also accounted for a significant proportion within the categories of “administrative issues” (33.3%, *n* = 8), “incomplete information” (38.1%, *n* = 8), and “dosage” (with 42.4% (*n* = 14) under-dosage and 35.7% (*n* = 5) over-dosage) given its role as an extremely broad-spectrum antimicrobial agent. Regarding DRPs caused by interactions, ciprofloxacin (25.0%, *n* = 8), linezolid (21.8%, *n* = 7), along with voriconazole were particularly notable.

### Pharmaceutical interventions

**Fig. 6:**
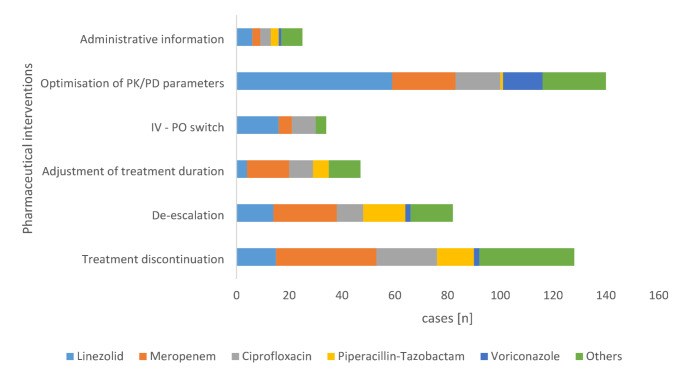
Pharmaceutical interventions (*n* = 456) triggered by restricted antimicrobials AST antimicrobial susceptibility testing; DRP drug-related problem; ID infectious diseases

The 456 pharmaceutical DRP-related interventions (Fig. 6) aimed at the optimization of pharmacokinetic/pharmacodynamic (PK/PD) parameters (*n* = 140, 30.6% of all interventions), treatment discontinuation (*n* = 128, 28.1%), and de-escalation (*n* = 82, 17.9%). Additionally, adjustment of treatment duration accounted for 10.3% of interventions (*n* = 47), while IV-to-oral (IV/PO) switch and administrative information by the pharmacists represented 7.4% (*n* = 34) and 5.5% (*n* = 25), respectively.

A clear pattern emerged between anti-infective agents and the pharmaceutical interventions they prompted. The optimization of PK/PD parameters—focusing on therapeutic drug monitoring, dose and dosing interval —was most frequently addressed for linezolid, which accounted for 42.1% of interventions in this category (*n* = 59), followed by meropenem (*n* = 24, 17.1%). Notably, linezolid also dominated interventions on IV-to-oral switching, representing 47% of consultations in this category (*n* = 16), followed by ciprofloxacin (26.5%, *n* = 9), both known for their high oral bioavailability. Meropenem was particularly prominent in consultations related to antimicrobial treatment discontinuation (*n* = 38, 29.7%) and adjustment of the treatment duration (34%, *n* = 16). Additionally, therapy de-escalation was most frequently required for treatments involving broad-spectrum agents, particularly meropenem (29.3%, *n* = 24) and piperacillin-tazobactam (19.5%, *n* = 16).

The proposed interventions were instantly implemented by the ward physicians in 82.7% of cases (*n* = 377), resulting in a change of the antimicrobial treatment following the pharmaceutical consultation. In 8.8% of cases (*n* = 40), the physician considered the pharmacist’s recommendation during consultation, but its final implementation was unclear, as the decision required discussion with a senior physician or additional patient information. Due to anonymized data, the retrospective analysis could not determine whether these recommendations were ultimately implemented. In 5.7% (*n* = 26), the implementations could not be directly assessed as they were classified as general informational or not applicable to a direct treatment modification. Only 2.8% (*n* = 13) of the interventions were rejected by the clinicians.

### Reasons for direct cost-savings by pharmaceutical interventions

Cost reduction potential was identified in 409 out of 456 pharmaceutical interventions (89.7%). Specifically, 33% (*n* = 151 interventions) were attributable to the reduction of ordered items (e.g. vials, drug packs) compared to the initial order, followed by drug discontinuation (15.5%, *n* = 71), and IV to oral switch (5.3%, *n* = 24). Reduction of the initial dose and switch to a less expensive antimicrobial agent played a minor role (Table 6). In 132 interventions (28.9%), a follow-up cost increase was anticipated due to a clinically indicated dose escalation, the necessity for therapeutic drug monitoring (e.g., linezolid, voriconazole), or additional laboratory and clinical assessments (e.g., electrolyte panels, ECG in patients with multiple cardiotoxic drugs).

**Table 6:** Interventions with cost-reduction potential in pharmacist-led post-prescription interventions of restricted antimicrobials (*n* = 456)

Cost-reduction potential	Interventions *n* (%)	
Reduction of order quantity	151	(33)
Follow-up costs	132	(28.9)
Drug discontinued	71	(15.5)
IV to oral switch	24	(5.3)
Dose reduction	13	(2,8)
Switch to a cheaper drug	18	(3.9)
Cost neutral change	21	(4.6)
Not related to costs	27	(5.9%)
**Total**	**457**	**(100 )**

The interventions implemented yielded potential cost savings of €180,420 through avoided anti-infective expenses (Table 7). Most of the cost savings were achieved through interventions involving meropenem (€50,866 in saved medication costs), linezolid (€33,154), anidulafungin (€17,851), ciprofloxacin (€17,149), and piperacillin-tazobactam (€11,185), which together accounted for 69% of the total anti-infective cost savings. As the pharmaceutical service required 0.25 FTE of a pharmacist, these interventions resulted in a return on investment of approximately 1:6, excluding additional effects from behavioral changes.

**Table 7:** Cost savings through pharmacist-led interventions post-prescription interventions of restricted antimicrobials (*n* = 456)

Substance	Savings € (%)	
Meropenem	50,866	(28.2)
Linezolid iv Linezolid po	27,869 5,285	(15.4) (2.9)
Anidulafungin	17,851	(9.9)
Ciprofloxacin iv Ciprofloxacin po	16,019 1,130	(8.9) (0.6)
Piperacillin-tazobactam	11,185	(6.2)
Caspofungin	7,998	(4.4)
Daptomycin	7,589	(4.2)
Posaconazole	4,906	(2.7)
Valganciclovir	4,678	(2.6)
Cefiderocol	4,500	(2.5)
Moxifloxacin iv Moxifloxacin po	3,514 118	(1.9) (< 1)
Ceftazidim-avibactam	3,360	(1.9)
Ertapenem	3,117	(1.7)
Voriconazole	2,523	(1.4)
Fidaxomicin	2,178	(1.2)
Fosfomycin iv	1,329	(< 1)
Levofloxacin iv Levofloxacin po	1,160 100	(< 1) (< 1)
Isavuconazole	806	(< 1)
Tigecyclin	741	(< 1)
Micafungin	607	(< 1)
Ceftriaxone	412	(< 1)
Amoxicillin-clavulanate	311	(< 1)
Rifampin	161	(< 1)
Trimethoprim-sulfamethoxazole	43	(< 1)
Ampicillin-sulbactam	24	(< 1)
Chlarithromycin	24	(< 1)
Erythromycin	19	(< 1)
**Total**	**180**,**420**	**100**

### Interrater reliability of clinical experts

The questionnaire response rate was 100%, with all seven experts completing and returning the questionnaire within 14 days. Table 8 (appendix) presents the questionnaire results regarding significance classification, including agreement with the original rating. No case exhibited a 100% agreement between the expert team and the clinical pharmacists from Salzburg. However, very high agreement levels of 84% and 85% were observed in two cases, while high agreement (70%) was found in four cases.

**Table 8:** Results of the questionnaire on clinical relevance rating

Results of the Questionnaire on clinical relevance rating									
		Clinical pharmacists				Physicians			Agreement with original rating
Questionnaire No.	Original rating	1	2	3	4	5	6	7	
1	4	3	4	3	3	3	4	3	28%
2*	0	0	0	0	1	2	0	---	68%
3	1	3	3	3	2	1	1	2	28%
4	1	0	1	1	2	2	1	2	42%
5	1	0	0	1	1	0	0	2	28%
6	1	0	1	1	1	2	2	2	42%
7	1	0	0	0	1	0	0	1	28%
8	3	4	4	2	2	2	3	4	14%
9	3	1	3	1	2	3	2	2	28%
10	3	2	3	2	2	2	2	1	14%
11	3	2	3	2	2	2	2	2	14%
12	3	1	4	3	3	3	2	3	56%
13	2	0	0	1	1	0	0	1	0%
14	2	2	3	2	2	3	2	2	70%
15	2	1	4	3	1	1	1	3	0%
16	2	2	4	3	3	2	3	3	28%
17	2	1	3	3	2	2	2	2	56%
18*	2	0	3	2	1	1	0	---	17%
19	2	3	2	2	2	2	2	1	70%
20	2	3	1	2	1	2	1	2	42%
21	2	0	0	1	0	0	0	1	0%
22	2	2	2	2	2	2	2	3	84%
23	2	3	3	3	2	2	2	2	56%
24	2	2	4	2	2	2	2	3	70%
25	2	3	3	3	2	3	2	3	28%
26*	2	2	3	2	2	---	2	2	85%
27	2	1	2	2	2	2	1	1	56%
28	2	2	2	2	2	3	1	2	70%
29*	2	4	3	2	2	2	2	---	56%
30	2	1	2	3	2	2	1	3	42%

* Questionnaire drop out due to missing data

The statistical analysis of interrater reliability was based on a sample size of *n* = 30 (except for the weighted Cohen’s kappa (*n* = 26) due to missing values) (Table 9, appendix). All Fleiss’ kappa coefficients indicate “slight agreement”. As expected, coefficients accounting for similar ratings (weighted Cohen’s kappa, AC2) are higher than those considering only exact matches (Fleiss’ kappa, AC1). Gwet’s coefficients also tend to be higher. Comparing clinical pharmacists and physicians, Fleiss’ kappa and weighted Cohen’s kappa indicate higher agreement among clinical pharmacists, whereas Gwet’s coefficients suggest stronger agreement among physicians. This discrepancy likely results from the coefficients’ sensitivity to unequal marginal distributions. Agreement between the expert team and the original clinical relevance rating (Table 10, appendix) is based on *n* = 30. Cohen’s kappa indicates “moderate agreement” for the entire expert team and clinical pharmacists, but only “slight agreement” for physicians. Across all coefficients, clinical pharmacists align more closely with the original clinical relevance rating than physicians.

**Table 9:** Coefficients for evaluating interrater reliability

Interrater-reliability of the expert team			
	Total	Clinical pharmacists	Physicians
Fleiss Kappa	0.176	0.198	0.100
Weighted Cohens Kappa	0.526	0.523	0.464
AC1	0.291	0.262	0.263
AC2	0.742	0.701	0.802

**Table 10:** Coefficients for expert team agreement with original rating of clinical relevance

Agreement of the expert team with the original rating of clinical relevance			
	Total	Clinical pharmacists	Physicians
Cohens Kappa	0.306	0.328	0.140
Weighted Cohens Kappa	0.596	0.558	0.500
AC1	0.487	0.485	0.370
AC2	0.872	0.868	0.850

## Discussion

Pharmaceutical post-prescription measures are fundamental components of AMS programs. Our study underscores the critical need for advisory services and interventions following anti-infective prescriptions in restricted antimicrobial agents. Additionally, we identified critical anti-infectives that warrant the most urgent stewardship efforts:

Among 3,897 prescriptions, 456 (11.7%) corresponding to 366 patients contained at least one DRP. Broad-spectrum antimicrobial linezolid (25%), meropenem (24%), ciprofloxacin (16%), and piperacillin-tazobactam (9%) were most often associated with pharmaceutical interventions, making up nearly 74% of all identified DRPs. Notably, 80.2% of the DRP-triggered interventions were classified of considerable clinical relevance based on an established rating scale. A subsample was cross-checked by external raters; they confirmed the relevance of interventions and showed fair to considerate agreement on the given grade. Further in-sights may require a larger sample size.

The primary causes of DRPs were due to non-conformance regarding either existing guidelines, ID-consultation advice or present AST (27.4% of all DRPs), and prescription without clear indication (27.2%), followed by a need for patient or therapeutic drug monitoring (12.5%). This unveils substantial deficiencies in the implementation of key aspects of therapeutic indication assessment and monitoring in routine clinical practice in our hospital. A closer analysis of those key antimicrobials revealed that linezolid-associated interventions were mainly due to inadequate therapeutic drug monitoring (82.4% of DRPs within this category), despite its availability and promotion in our hospital. In addition, the substance was often prescribed without a clear indication (16.9%), or there was no conformance with guidelines, existing ID consultation or microbiological findings (16%). The situation with meropenem was equally concerning: With this substance a significant amount of dosage errors were observed (42.4% underdosing and 35.7% overdosing within this category), and meropenem was the most commonly involved substance regarding a lack of clear indication (32.2%) and non-compliance with guidelines, ID consultation, or the available AST (24.0%). Additionally, issues such as missing prescription details (38.1%), and failure to administer the drug (33.3%) were prevalent. These findings are particularly concerning, given that meropenem is considered a reserve antibiotic, which underscores the need for strict adherence to guidelines and proper usage.

Our findings align with existing literature that addresses the high prevalence of DRPs. Over- and misuse of antimicrobial agents are common, especially with restricted and broad-spectrum agents such as meropenem [2]. A recent multicenter study in non-university hospitals revealed low and variable adherence to antimicrobial prescribing quality indicators, with poor compliance in documentation, streamlining, and IV-to-PO switching [16].

Here identifying anti-infective agents requiring intervention is a key step in improving antimicrobial management. White et al. demonstrated that pre-authorization reduced total antibiotic use by 32% and decreased resistance in Gram-negative bacilli [17]. However, these measures must be considered under a certain behavioral change perspective and cannot be addressed independently. A 2013 Cochrane review of 52 interrupted time series further showed that restrictive and persuasive interventions lower resistance and unnecessary antimicrobial use, emphasizing the need for training and behavioral change [18]. More recently, Okihata et al. [19] found that continuous education and expert guidance in a Japanese dental university clinic reduced third-generation cephalosporin use and improved prescribing practices.

Restrictive antimicrobial use and post-prescription review should be considered in the broader context of multifaceted antimicrobial stewardship efforts, rather than as standalone measures. Our own research in diagnostic stewardship has demonstrated that microbiological results are frequently overlooked on the ward, resulting in inappropriate anti-infective use due to limited awareness and suboptimal initial sample collection. In this context, misuse was particularly common with broad-spectrum antibiotics such as meropenem, piperacillin-tazobactam, and ciprofloxacin, underscoring the pivotal role of the microbiology laboratory in optimizing antimicrobial therapy [20]. A comprehensive stewardship strategy incorporates also diagnostic measures—such as point-of-care and syndromic multiplex PCR testing—to reduce unnecessary anti-infective therapies. For example, a multiplex PCR panel for respiratory infections prompted therapy adjustments in 70.7% of cases, mainly through de-escalation of vancomycin (38%) and piperacillin-tazobactam (23%) [21]. However, as Keske et al. (2018) demonstrated, these tests do not fully prevent inappropriate antibiotic use, emphasizing the need for enhanced clinician training in interpreting and applying test results [22].

Key drawbacks of the pre-authorization process of restricted antimicrobials are a reduced prescriber autonomy and the time-intensive nature of pre-authorization, potentially causing treatment delays and physician dissatisfaction. Restricting anti-infective agents also requires caution, as it may shift usage to non-restricted agents (“squeezing the balloon” effect) [8]. Rahal et al. demonstrated this when cephalosporin pre-approval reduced ceftazidime-resistant *Klebsiella* species but increased carbapenem use, leading to a 69% rise in *Pseudomonas aeruginosa* resistance [23]. Thus, a precise, real-time surveillance system for both restricted and unrestricted antimicrobials is essential to prevent unintended shifts in prescribing practices. Effective implementation requires early coordination among clinical pharmacists, ward physicians, and infectious disease specialists, as well as broad consensus involving hospital management, IT departments, and software providers [8].

Pharmacist-led interventions can achieve notable cost savings by reducing daily drug requirements and shortening hospital stays, particularly when integrated with TDM [24]. Avoided costs of €180,420 in the present study highlight the economic impact of pharmacists’ interventions. To further optimize antimicrobial use, we propose streamlining hospital guidelines, clarifying microbiological diagnostic requirements, enhancing TDM services, improving prescriber education, strengthening monitoring, and integrating digital tools for automated DRP detection and real-time alerts.

### Limitations

Several limitations should be acknowledged. Outside regular working hours, pharmacists without AMS training were more likely to dispense restricted antimicrobials without reviewing patient records and tended to approve their use more liberally than AMS-trained colleagues. This may reflect gaps in specialized knowledge and pressure to expedite dispensing during on-call shifts. Additionally, as a secondary data analysis, the study relied on routine documentation not originally intended for research purposes. Data were entered by three pharmacists following the same guidelines, yet interpretation of drug-related problems and interventions may have varied. Cost-savings were calculated based solely on non-dispensed items; potential substitutions from one substance to another were not accounted for in the cost comparison.

## Conclusion

This study highlights the essential role of clinical pharmacists in antimicrobial stewardship (AMS), particularly in addressing drug-related problems (DRPs) involving high-risk and broad-spectrum agents. Non-adherence to guidelines, as well as unclear indications, emerged as key reasons for inappropriate antimicrobial requests. By integrating pharmacist-led post-prescription reviews with structured DRP categorization and agent-specific profiling, we provide actionable insights for targeted AMS interventions and benchmarking. The clinical relevance of our interventions was externally validated, and real-world cost savings were demonstrated. An acceptance rate of 82.7% confirms the effective incorporation of pharmacists into AMS teams. These findings emphasize the need for strengthened AMS measures such as de-escalation, TDM-based dosing, and IV-to-oral switching. Moreover, we encourage other AMS teams to critically evaluate their stewardship structures, as the outlined elements are essential for achieving a comprehensive and holistic understanding of AMS systems. Future research should explore the underlying causes of antimicrobial overuse and misuse as well as barriers to optimal prescribing behaviors.

## Data Availability

No datasets were generated or analysed during the current study.
